# Two-transcript signature for differentiation and clinical outcomes in severe fever with thrombocytopenia syndrome (SFTS) patients: a double-blind, multicenter, validation study

**DOI:** 10.1128/jcm.01282-24

**Published:** 2024-12-17

**Authors:** Nannan Xu, Sai Wen, Yongyuan Yao, Yanyan Guan, Lianhui Zhao, Lulu Yang, Hui Yang, Yishan He, Gang Wang

**Affiliations:** 1 Department of Infectious Disease, Qilu Hospital, Cheeloo College of Medicine, Shandong University 66555 , Jinan, Shandong, China; 2 Juxian People’s Hospital, Rizhao, China; 3 Department of Infectious Disease, Rizhao People's Hospital 549615 , Rizhao, China; Wadsworth Center - NYSDOH, Albany, New York, USA

**Keywords:** severe fever with thrombocytopenia syndrome, diagnosis, host transcript, severity

## Abstract

Severe fever with thrombocytopenia syndrome (SFTS) is an emerging infectious disease with a high mortality rate that is often underdiagnosed due to the limitations of current laboratory testing. Timely diagnosis and early identification of severe cases are crucial to improving patient outcomes and overall survival rates. This study aimed to evaluate the efficacy of two transcripts, IFI44L and PI3, in the early differentiation between SFTS virus (SFTSV) infection and bacterial sepsis, as well as in the prompt identification of severe cases during epidemic seasons. In a prospective study conducted between 1 May 2021 and 30 September 2022, we enrolled 225 patients who presented with acute fever and thrombocytopenia at four hospitals in Shandong Province, China. The two-transcript signature provided a clear distinction between SFTS and bacterial infection, achieving an area under the receiver operating characteristic curve of 0.961 (95% confidence interval [95% CI] 0.916–0.986), outperforming C-reactive protein (0.810 [95% CI 0.738–0.870]) and procalcitonin (0.764 [95% CI 0.687–0.830]). Importantly, the relative expression of the IFI44L gene was significantly elevated in fatal SFTS cases, with an area under the curve (AUC) of 0.820 (95% CI 0.727–0.914), indicating its potential as an early prognostic marker. Additionally, IFI44L and PI3 were identified as potential biomarkers for distinguishing SFTS patients with and without invasive pulmonary aspergillosis, with AUC values of 0.817 and 0.753, respectively. Our findings demonstrate that the two-transcript signature effectively distinguishes SFTSV infection from bacterial sepsis and helps identify high-risk individuals, guiding appropriate treatment during SFTS outbreak.

## INTRODUCTION

Severe fever with thrombocytopenia syndrome (SFTS) is an emerging tick-borne infectious disease caused by the SFTS virus (SFTSV), which belongs to the genus *Bandavirus* in the family *Phenuiviridae*, order *Bunyavirales*. First isolated in central China in 2009, SFTS has since spread to multiple provinces in East China (1), and has been reported in many countries worldwide, including the United States, South Korea, Japan, and Vietnam (2, 3). Data from the Chinese CDC indicate that the number of regions affected by SFTS is steadily increasing in China, with the number of cases increasing annually (2). In addition to transmission through tick bites, SFTSV can also spread via contact with the body fluids of infected patients, with the oral and ocular membranes serving as potential routes of entry (4, 5). The most common clinical manifestations are an abrupt onset of fever, thrombocytopenia, leukocytopenia, and respiratory tract or gastrointestinal symptoms (6). Despite regional variations, the mortality rate remains relatively high, ranging from 12% to 30% (7). Furthermore, a significant number of SFTS patients develop invasive pulmonary aspergillosis (IPA), which is directly linked to a high fatality rate (8).

Although SFTS is endemic, misdiagnosis can still occur, particularly when symptoms of acute fever and thrombocytopenia overlap with other conditions. Bacterial sepsis is a common misdiagnosis, and the uncertainty of its diagnosis can lead to the unnecessary use of antibiotics and poor prognosis. Therefore, early identification of SFTS is crucial for optimal resource allocation, clinical improvement, SFTS prevention and control, and reducing the risk of antibiotic resistance. Currently, the diagnosis of SFTS is based on serological methods and polymerase chain reaction (PCR)-based viral nucleic acid testing, with PCR considered the gold standard. However, the positive rate of PCR has been reported to be only 50% (9). Serological methods have low specificities, and specific antibodies may be negative during the early stage of the disease. Therefore, the laboratory diagnostic rate is only 64.4% (3). This means that more than 30% of suspected SFTS patients could not be confirmed by laboratory tests (10, 11), which causes the actual incidence of SFTS to be underestimated (12).

Recognizing these challenges, our study sought to explore the complementary role of host transcriptional analysis in the early differentiation of SFTS, with a specific focus on identifying fatal cases. Many studies have indicated that the host immune response is a significant factor related to the severity of diseases, including sepsis (13, 14), dengue (15), severe coronavirus disease 2019 (COVID-19) (16), and other diseases (17). In our prior study, we revealed the discovery and validation of two transcripts—a signature based on the whole-blood expression levels of the IFI44L and PI3 genes, which are integral to the host response to infection (18). These two-transcript biomarkers have exhibited promise in distinguishing patients with bacterial and viral infections, with an overall area under the curve (AUC) of 0.969. Nonetheless, the efficacy of these transcripts in the early differentiation of SFTS and identification of fatal cases remains uncertain.

In this study, our primary objective was to assess the potential of these two transcription biomarkers for the early differentiation of SFTSV infection and the prompt identification of severe cases during epidemic seasons.

## MATERIALS AND METHODS

### Study design and participants

**Fig 1:**
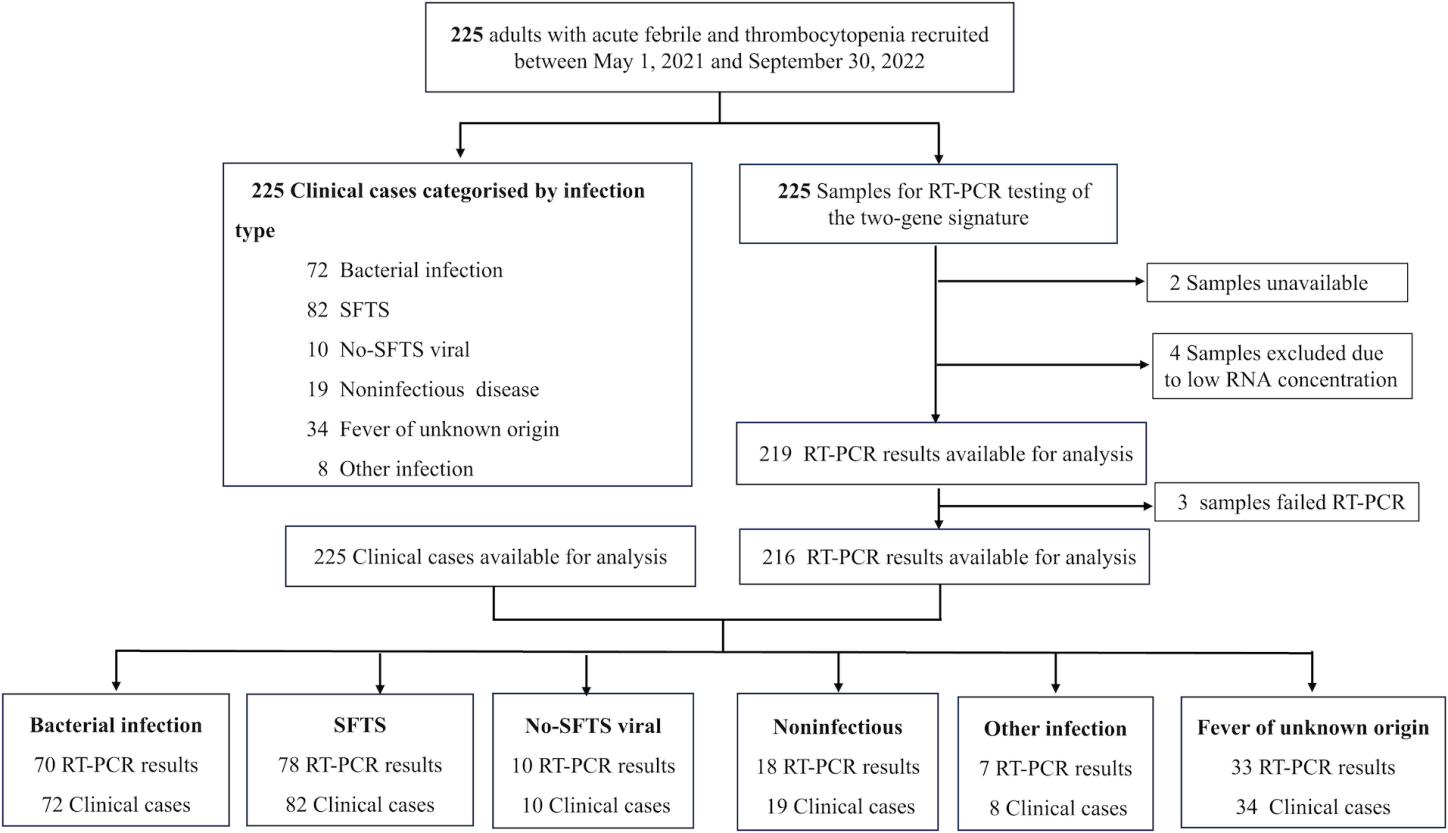
Study profile.

Patients who presented between 1 May 2021 and 30 September 2022 to the fever clinic or infectious diseases department of Shandong University Qilu Hospital, Rizhao People’s Hospital, Linyi Central Hospital, and Juxian People’s Hospital, with suspected SFTS, were prospectively screened for eligibility. Patients were considered eligible if they were 18 years or older; had clinical manifestations (acute fever [axillary temperatures ≥38°C]), leukocytopenia (<4.0 × 10^9^ /L), and thrombocytopenia (<100 × 10^9^/L); and had an epidemiologic history (i.e., working, living, or traveling in hills, forests, or mountains within the SFTS epidemic season or being bitten by ticks within 2 weeks before the disease onset) (19). Patients were not eligible if they had symptoms for more than 14 days, or had comorbidities (such as active malignancy, primary or secondary immunodeficiency, or taking immunosuppressants), pregnancy, mixed infection, or incomplete clinical information. The eligibility and workflow of this study are summarized in Fig. 1.

### Procedures

Within 24 hours of admission, all patients received routine examinations. Except for blood cultures and blood draws for two transcripts, other routine laboratory evaluations were performed by the treating physician. These typically include blood cell count and differential, procalcitonin (PCT), C-reactive protein (CRP), blood chemistry, urinalysis, and urine culture, among others. Computed tomography (CT) was performed on the basis of clinical indications. The physicians and nursing staff involved in the treatment of patients were unaware of the transcript test results. The two-transcript test was used to evaluate the expression levels of the IFI44L and PI3 genes. A predefined logistic regression formula was used to calculate the likelihood score for determining a bacterial or viral infection, as previously described (18), and further details can be found in the supplementary materials. On the basis of the likelihood score of the two-transcript test, each patient was classified as having a bacterial or a viral immune response. Patients with a two-transcript test score above 0.547598 were classified as having bacterial infections, whereas those with a score below 0.547598 were classified as having viral infections. The smaller the value is, the greater the likelihood of a viral infection.

Clinical data were gathered from medical records via an electronic case report form (eCRF). The eCRF included demographic information, medical history, comprehensive radiological and laboratory data collected during routine care (such as procalcitonin levels, white blood cell counts, and absolute neutrophil counts), microbiological investigation outcomes (including blood culture and reverse transcription-PCR (RT-PCR) for SFTSV specific to the study), and follow-up details. Two clinicians who were blinded to the two-transcript test results assigned one of the following causes to each patient: bacterial infection, SFTSV infection, no-SFTS viral infection, noninfectious disease, or indeterminate.

### Gene expression analysis

Host gene expression was measured as previously described (18). In brief, blood samples were drawn from the patients and placed into PAXgene tubes (Qiagen, Valencia, CA). Total RNA was extracted using the PAXgene Blood RNA Kit according to the manufacturer’s protocol. All the extracted RNA samples were reverse-transcribed into cDNA using a reverse-transcription kit (Sangon Biotech Corp.) according to the manufacturer’s instructions. The obtained cDNA was amplified via the Hongshi SLAN96P real-time detection system with the TaqMan Mix (Sangon Biotech Corp.). The TaqMan PCR kit and the primers for the target genes, including IFI44L (Hs00915292_m1), PI3 (Hs00160066_m1), and the internal control ACTB (Hs01060665_g1), were purchased from Applied Biosystems. The quantitative PCR (qPCR) assays were performed in duplicate, and the relative expression levels were normalized to those of the reference gene (ACTB) via the ΔCt method (20).

### Detection of SFTSV RNA in acute-phase serum samples

A total of 200 µL of serum was used for RNA extraction via the RNeasy Mini Kit (Qiagen, Germany). The SFTSV nucleic acid tests were performed via an SFTSV Real Time RT-PCR Kit (Daan gene, Guangzhou, China) according to the manufacturer’s protocol. The reaction parameters were 50°C for 15 min, 95°C for 15 min, and then 45 cycles of 95°C for 15 s and 55°C for 45 s. The cutoff cycle threshold (Ct) value was set at 35 cycles. The copy number of viral cDNA in copy number per milliliter serum samples was determined by comparison with a serially diluted plasmid standard of known concentration.

### Definition of IPA among SFTS patients

To analyze the pathogenesis of SFTS with IPA, IPA was regularly evaluated during hospitalization. We defined aspergillosis in patients with SFTS using a modified IPA algorithm as described previously (21). Briefly, the IPA definition was based on clinical, radiological, and mycological criteria as the presence of the following three criteria: compatible signs and symptoms of IPA, abnormal findings on either a chest X-ray or CT scan of the lungs, and mycological criteria such as histopathological evidence of hyphae with recovery of *Aspergillus* from tissue, positive culture from a bronchoalveolar lavage (BAL), a galactomannan (GM) index in in BAL fluid ≥1.0, or single serum or plasma ≥1.0, or single serum or plasma ≥0.7 and BAL fluid ≥0.8.

### Statistical analysis

SFTSV RNA concentrations (copies per milliliter) were log-transformed before statistical analyses were performed. Categorical variables are presented as frequencies and percentages, whereas continuous variables are presented as medians and interquartile ranges (IQRs). The Kolmogorov-Smirnov test was used to inspect the normality and homogeneity of the variance of all of the data. The baseline features between the two groups were compared via the *χ*^2^ test (categorical variables), Student’s *t*-test, or the Mann-Whitney *U*-test. An area under the receiver operating characteristic curve (AUROC) was used to evaluate the performance of two transcripts for the early diagnosis and identification of fatal SFTS patients. The 95% confidence intervals (95% CIs) of those values were assessed via the Wilson score algorithm. The difference in the AUCs between pairs of receiver operating characteristic (ROC) curves was evaluated with DeLong’s test (22). A likelihood score cutoff of 0.547598, a PCT cutoff of 0.5 ng/mL, and a CRP cutoff of 30 mg/L were used to calculate the accuracy, sensitivity, specificity, positive predictive value, negative predictive value, positive likelihood ratio, and negative likelihood ratio. Spearman’s rank correlation coefficient was calculated to assess correlations between viral concentration and laboratory evaluation. A *P*-value of <0.05 was regarded as statistically significant. All statistical analyses were performed with SPSS (version 24.0), GraphPad Prism (version 8.0), and MedCalc (version 18.2.1).

## RESULTS

### The performance of a two-transcript signature in differentiating SFTS from bacterial infections

Between 1 May 2021 and 30 September 2022, 225 consecutive participants with suspected SFTS were enrolled from Shandong University Qilu Hospital, Rizhao People’s Hospital, Linyi Central Hospital, and Juxian People’s Hospital. According to the diagnostic categorization algorithm (Fig. S1), 82 (36.4%) patients had a final diagnosis of SFTS, 72 (32.0%) patients had bacterial sepsis, 10 had non-SFTS viral infection, 34 had fever of unknown origin, 19 had noninfectious diseases, and eight had other alternate infections (Fig. 1). From the 225 participants in our study, RNA samples for the two-transcript RT-qPCR testing were unavailable from two participants, RNA concentrations were too low in four, and three samples failed RT-qPCR (Fig. 1).

To evaluate the hypothesis that the two-transcript signature can differentiate between bacterial infections and SFTS, we analyzed blood RNA samples from 72 participants with bacterial sepsis and 82 participants with SFTS. All participants, except four in the SFTS group and two in the bacterial group, had analyzable RT-qPCR results. The median time from symptom onset to admission was 8 (6–10) days. Compared with patients with bacterial infections, patients with SFTS tended to have lower white blood cell (WBC) count, neutrophils (NEU), CRP, PCT, blood urea nitrogen (BUN), prothrombin time (PT), and fibrinogen levels. However, the lymphocytes (LYM), hemoglobin (HGB), ferritin, aspartate aminotransferase (AST), albumin (ALB), lactic dehydrogenase (LDH), and activated partial thromboplastin time (APTT) were greater in SFTS than in bacterial infection. Platelet (PLT), alanine aminotransferase (ALT), creatinine (Cr), and D-dimer (DDI) did not differ significantly between SFTS and bacterial infections. The demographic and clinical characteristics of the SFTS and bacterial infection groups are described in Table S1.

**Fig 2:**
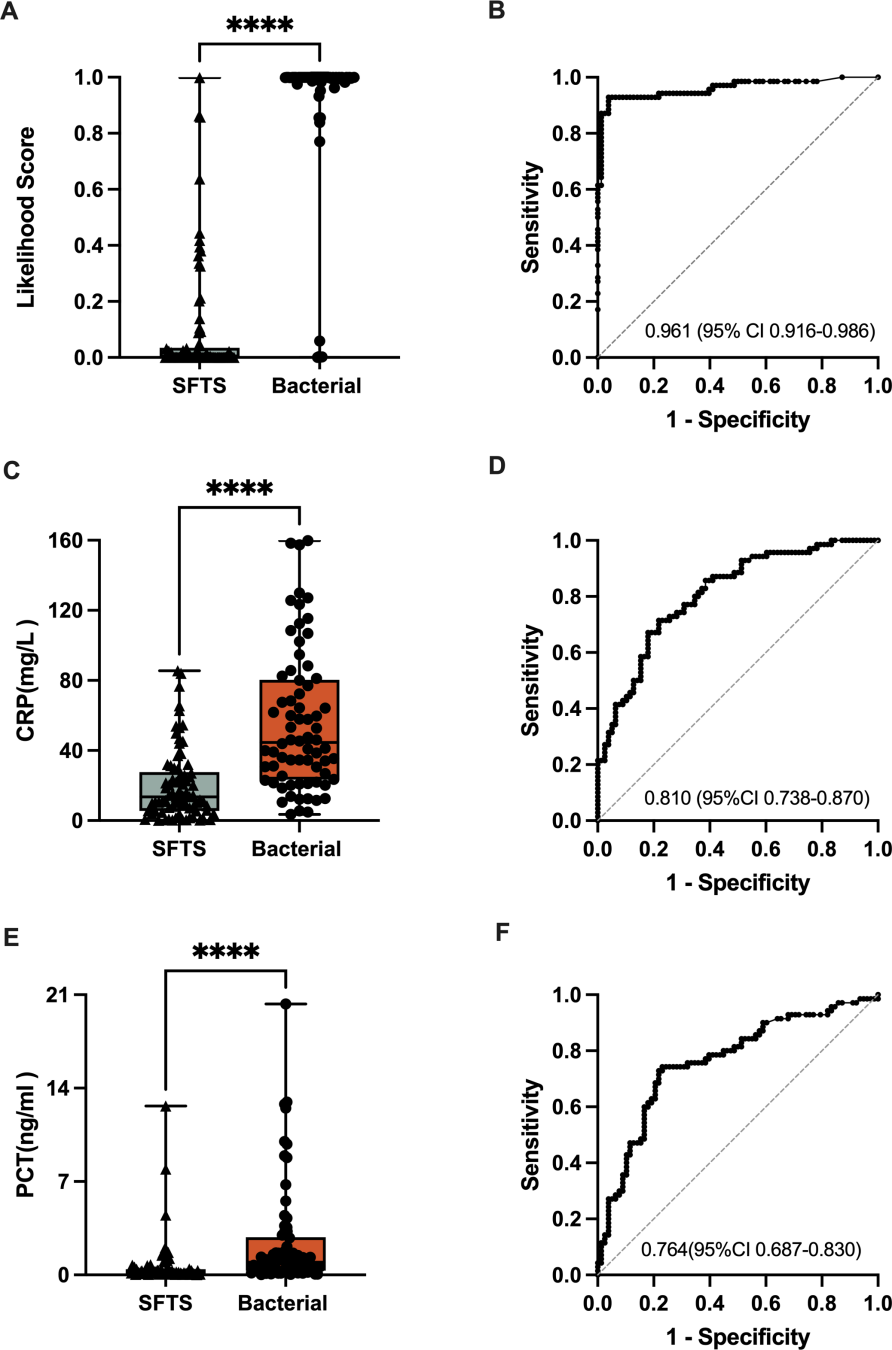
Performance of the two-transcript signature in the prospective validation cohort. Boxplots showing the two-gene signature (**A**),CRP (**C**),and PCT (**E**)in the prospective validation cohort comparing different infection categories. ROC curves of the two-transcript signature (**B**),CRP (**D**),and PCT (**F**)for bacterial versus SFTS comparison. Boxplots show the means and IQRs. AUC, area under the curve; CRP, C-reactive protein; PCT, procalcitonin; ROC, receiver operating characteristic; **** indicates a *P*-value <0.0001.

The two-transcript signature provided a clear distinction between SFTS and bacterial infection, with an AUROC of 0.961 (95% CI 0.916–0.986)(Fig. 2). The sensitivity and specificity of the two-transcript test for differentiating SFTS were 0.929 (95% CI 0.843–0.969) and 0.949 (95% CI 0.875–0.980), respectively, from bacterial infection. CRP and PCT achieved an AUROC of 0.810 (95% CI 0.738–0.870) and 0.764 (95% CI 0.687–0.830),(Fig. 2) respectively; the sensitivity and specificity were 0.714 (95% CI 0.600–0.807) and 0.769 (95% CI 0.664–0.849), respectively, for CRP, and 0.714 (95% CI 0.560–0.807) and 0.782 (95% CI 0.678–0.859), respectively, for PCT. The overall performance of the two-transcript signature, CRP and PCT, in distinguishing SFTS from bacterial infection is summarized in Table S2. When the SFTS group is merged with other viral groups, the two-transcript test achieves an AUROC of 0.960 (95% CI 0.926–0.994) in distinguishing viral infections from bacterial infections (Fig. S2).

For participants with noninfectious diseases and fever of unknown origin, the signature produced mixed results, as anticipated (see Fig. S2).

### Differences in the clinical parameters and expression of the two transcripts between SFTS survivors and fatalities

At follow-up, 18 of the 78 patients had died from the current episode of SFTSV infection, resulting in a fatality rate of 23.1%. The SFTS patients were categorized into “surviving” or “fatal” groups on the basis of their final clinical outcomes. There were no significant differences in age, sex, or time from onset to hospital stay between the two groups. The patients who died had significantly higher levels of NEU (2.43 vs 1.65 × 10^9^/L), CRP (41.29 vs 10.73 mg/L), PCT (1.012 vs 0.132 ng/mL), ferritin (8,467 vs 2,933 ng/mL), AST (360 vs 122 U/L), Cr (93 vs 63 µmol/L), BUN (9.9 vs 5.1 mmol/L), LDH level (1,094 vs 615 mmol/L), prolonged PT (13.6 vs 12.5 second), APTT (49.4 vs 39.0 second), and DDI (2.75 vs 1.87 µg/mL). ALB (31 vs 33 g/L) was lower in the fatal group than in the survival group. No significant difference was observed between the two groups regarding the WBC count, LYM, HGB, PLT, ALT, or fibrinogen levels. Detailed information on the demographic and clinical characteristics of the patients is shown in Table S3.

**Fig 3:**
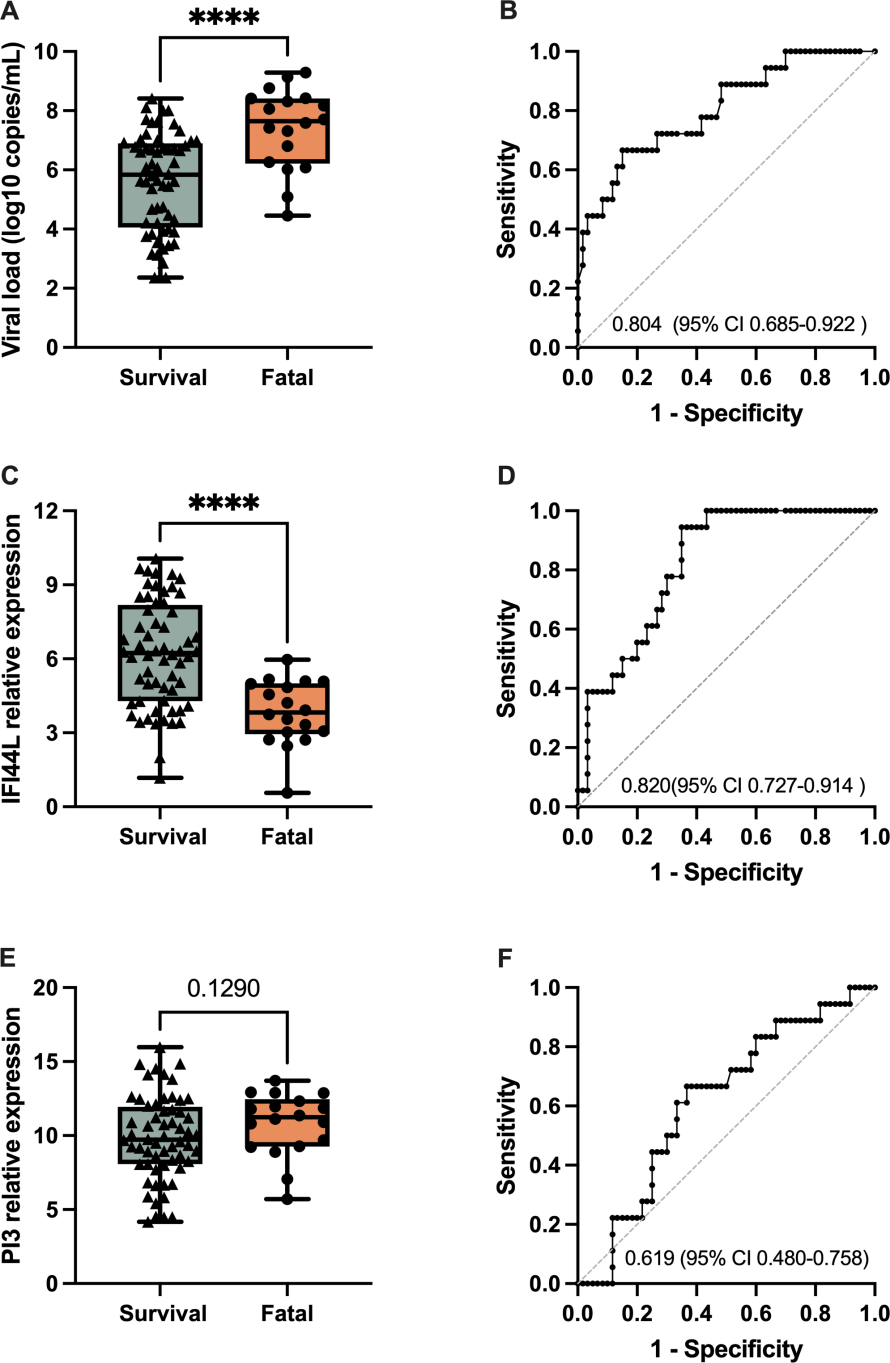
Performance of IFI44L and PI3, compared with viral load, as the predictors of prognosis. The relative expression levels (left) and the ROC curves (right) of each gene are displayed. SFTSV RNA concentrations (copies per milliliter) were log-transformed. The relative expression of each gene was displayed as delta Ct values. In the left panels, the horizontal lines in boxes indicate medians; the top and bottom ends of the boxes indicate the IQRs; and the upper and lower error bars extend to 1.5× the IQRs. Each dot represents an individual sample. **** indicates a *P*-value < 0.0001. Boxplots showing the viral load (A), IFI44L expression levels (C), and PI3 expression levels (E) in the prospective validation cohort, comparing survival and fatal groups. Receiver operating characteristic (ROC) curves for viral load (B), IFI44L (D), and PI3 (F) demonstrate their ability to distinguish between ssurvival and fatal groups.

Viral replication and host immune responses affect the severity and clinical outcome of SFTS (23). The viral RNA concentrations ranged from 2.29 × 10^2^ to 1.95 × 10^9^ copies per milliliter of serum, and significantly greater viral loads were detected among the patients who died (viral load log10-transformed 7.65 [IQR 6.22–8.41] copies per milliliter; *P* < 0.001) than among those who survived (viral load log10-transformed 5.84 [IQR4.06–6.90] copies per milliliter) (Fig. 3A). Next, we tested the performance of the *IFI44L* and *PI3* genes in distinguishing fatal cases from SFTS patients. Compared with that in patients who survived, the relative expression of IFI44L was significantly upregulated (*P* < 0.001) in patients who died (Fig. 3C), where a lower delta CT value indicates a high expression level. However, no significant difference in the relative expression of PI3 was detected between surviving and fatal patients (Fig. 3E). The AUC of the ROC curve for the viral load, a widely recognized prognostic factor for SFTS (24), was 0.804 (with a 95% CI of 0.685–0.922, *P* < 0.001). Specifically, the AUC for IFI44L was 0.820 (95% CI 0.727–0.914, *P* < 0.001). There were no statistically significant differences in the AUCs between IFI44L and viral load. These results highlight the remarkable performance of IFI44L as a potential biomarker and underscore its promising utility in establishing an effective early warning system for fatal cases of SFTS.

We analyzed the SFTSV viral load and IFI44L and PI3 expression levels to determine their relationships. Notably, the expression level of IFI44L (in terms of the delta CT values) was positively correlated with the viral load (*r* = −0.532, *P*-value <0.001; Fig. S3A); a lower delta CT value indicates a high expression level. There was no correlation between the expression level of PI3 and the viral load (*r* = 0.190, *P* = 0.095; Fig. S3B). This positive relationship could indicate that as the virus replicates more extensively, the host immune system responds by upregulating IFI44L, thereby enhancing antiviral defenses.

### Differences in the clinical parameters and expression of the two transcripts between SFTS patients with or without IPA

In our study, IPA was identified in 50% (39/78) of the SFTS patients, with a notably higher complication rate than that reported in previous studies, which ranged from 20% to 31.9% (8, 25). We further analyzed the clinical and laboratory characteristics of SFTS patients with or without IPA. Interestingly, there were no discernible differences in age, sex, or duration of illness since onset at admission between the two groups. However, a detailed examination of clinical laboratory parameters revealed significant elevations in WBC, NEU, CRP, PCT, ferritin, ALT, AST, LDH, BUN, Cr, and APTT in patients with IPA. Additionally, a trend toward lower PLT and ALB was observed in IPA patients. The demographic and clinical characteristics of the patients are presented in detail in Table S4.

**Fig 4:**
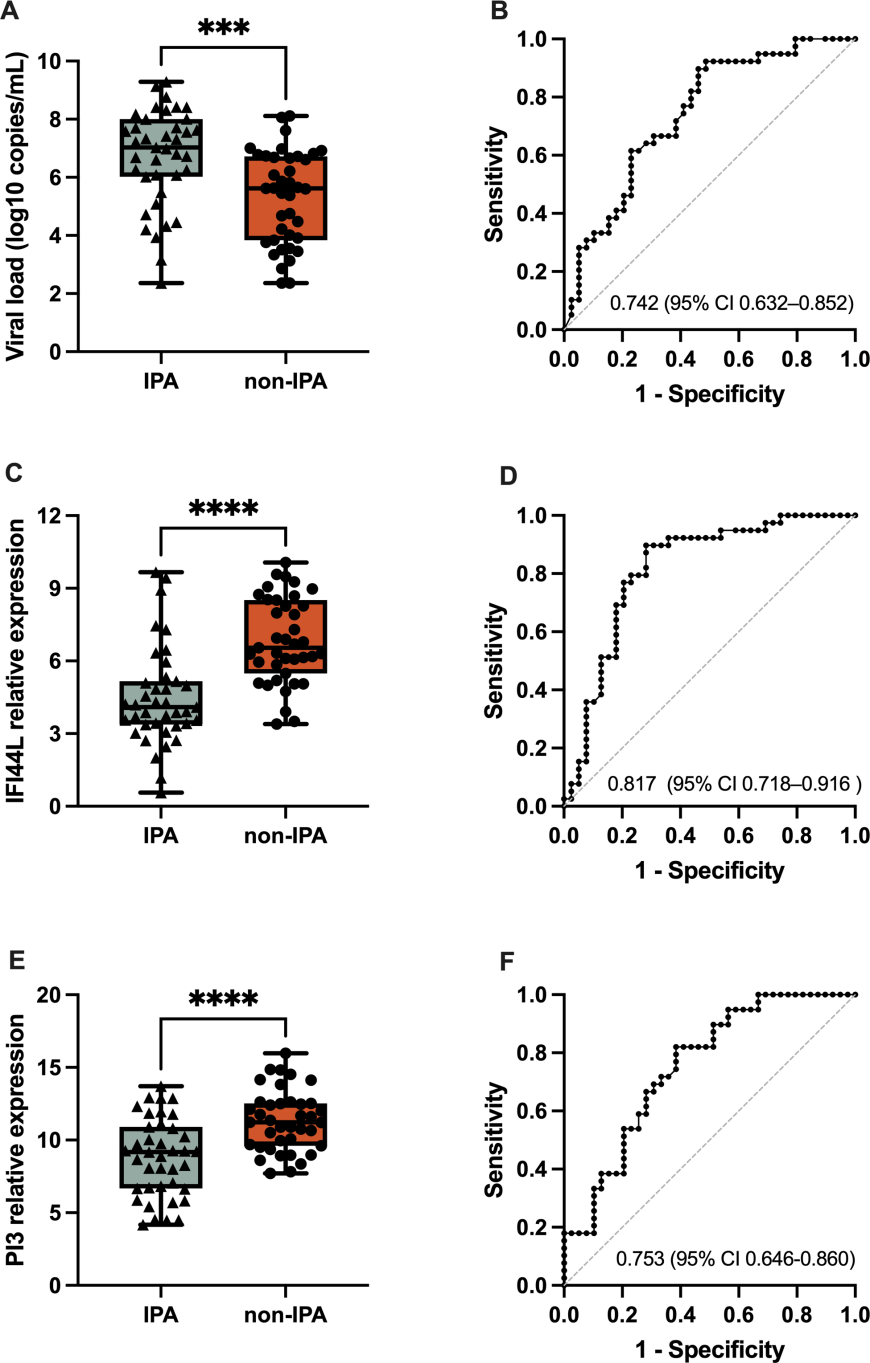
Performance of the IFI44L and PI3, compared with viral load, as the predictors of secondary invasive pulmonary aspergillosis. The relative expression levels (left) and the ROC curves (right) of each gene are displayed. SFTSV RNA concentrations (copies per milliliter) were log-transformed. The relative expression of each gene was displayed via delta Ct values. In the left panels, the horizontal lines in the boxes indicate medians; the top and bottom ends of the boxes indicate the IQRs; and the upper and lower error bars extend to 1.5× the IQRs. Each dot represents an individual sample. **** indicates a *P*-value <0.0001; *** indicates a *P*-value <0.001.1. Boxplots depicting the viral load (A), IFI44L expression levels (C), and PI3 expression levels (E) in IPA and non-IPA groups. Receiver operating characteristic (ROC) curves for viral load (B), IFI44L (D), and PI3 (F) demonstrate their effectiveness in distinguishing between these two groups.

We subsequently assessed the viral load and the levels of the IFI44L and PI3 genes in SFTS patients with and without IPA. Notably, the concentrations of viral RNA were markedly greater in patients with IPA than in those without IPA (Fig. 4A). Furthermore, the relative expression of IFI44L was significantly greater (*P* < 0.001) in patients with IPA than in patients without IPA (Fig. 4C). Similarly, the relative expression of PI3 was also significantly elevated (*P* < 0.001) in patients with IPA, as depicted in Fig. 4E. These observations underscore the potential association between IPA and increased viral replication, as well as the upregulation of IFI44L and PI3 gene expression, shedding light on the intricate molecular dynamics in SFTS patients with invasive fungal complications.

The AUC of IFI44L for distinguishing IPA patients from patients with SFTS was 0.817 (95% CI 0.718–0.916, *P* < 0.001), and the AUC for PI3 was 0.753 (95% CI 0.646–0.860, *P* = 0.0001). Although the AUC for the viral loads (0.742, 95% CI 0.632–0.852; *P* = 0.0002) was lower than those of IFI44L and PI3, the differences in the AUCs were not statistically significant. These results suggest that viral load, IFI44L, and PI3 could serve as potential biomarkers for distinguishing between SFTS patients with and without IPA.

## DISCUSSION

The emergence of SFTSV infection has become a significant public health concern in China. Early diagnosis of SFTS and identification of potentially fatal cases are essential for effective patient triage and resource allocation. Our study demonstrated that the blood transcripts of two specific genes provide an effective method for distinguishing SFTS from bacterial infection, with an AUROC of 0.961, a sensitivity of 0.929, and a specificity of 0.949. Notably, these two genes could also serve as potential biomarkers for identifying patients with fatal SFTS and as reliable predictors of complications by IPA.

Early identification of predictors for fatal SFTS cases is crucial for reducing mortality, enabling efficient patient triage and resource allocation, especially during peak SFTS periods. While high viral load is a well-established predictor of poor outcomes (26–28), the acquisition of viral load and differences between assays may impact predictive accuracy. Various signatures using clinical variables have been proposed for detecting severe SFTS cases (29, 30); however, except older age and high viral load, other risk factors show discrepancies in their association with death. Therefore, identifying a host response signature holds promise for enhancing our understanding of SFTS pathogenesis and identifying individuals at greater risk of severe outcomes.

In our study, IFI44L emerged as a potential biomarker for the early identification of fatal SFTS, with an AUC of 0.820. IFI44L, an interferon (IFN)-stimulated gene (ISG) induced by various viruses (31–33), acts as a feedback regulator of IFN responses, potentially supporting viral replication (34). Its upregulation in BAL cells and peripheral blood mononuclear cells of patients with severe COVID-19 suggests a role in predicting disease severity (16). In our study, fatal SFTS patients presented increased IFI44L expression, which was correlated with viral load. An excessive IFN-I response could lead to a cytokine storm, worsening the patient’s condition (35). These findings indicate that in severe SFTS patients, excessive activation of the IFN pathway results in heightened inflammatory responses, severe immunopathological damage, and fatal outcomes. Interrupting the “cytokine storm” could alleviate the severity of the disease. These findings provide potential targets for anti-inflammatory treatment strategies for SFTSV infection. Currently, there are reported cases of severe SFTS treated with immunomodulatory drugs, such as interleukin (IL)-6 inhibitors (36) and Janus kinase (JAK) inhibitors (37). IFI44L may be a useful clinical marker for monitoring the early progression of severe SFTS and could be a potential target for SFTS intervention. In addition, we found that patients with high IFI44L expression in the early stage of disease were more likely to develop complications from IPA. IFI44L may also be linked to immune dysregulation, contributing to secondary fungal infection.

The immune dysregulation observed in severe SFTS patients is closely linked to susceptibility to fungal infections, with 50% of SFTS patients experiencing IPA in our study. The mechanisms of secondary IPA in SFTS patients remain unclear, but immunity to fungal diseases relies on both humoral and cellular responses (38). SFTSV infection simultaneously disrupts both the innate and adaptive immune systems, increasing the susceptibility of patients to IPA (39, 40). Furthermore, viral infection-associated defects in neutrophil functions can reduce host resistance against fungal pathogens (41). For example, during influenza infection, the virus can hinder neutrophil recruitment and phagocytosis, increasing vulnerability to *Aspergillus* infection (42). Similarly, severe COVID-19 pneumonia prompts blood neutrophils to adopt an antiviral state, possibly compromising their ability to combat fungi (43).

The PI3 gene encodes two proteins, tappin-2/pre-elafin and elafin, which demonstrate antimicrobial properties against bacteria, fungi, and viruses (44). These proteins play crucial roles in inflammation, innate immunity, and vascular remodeling, effectively preventing excessive tissue damage induced by neutrophils and macrophages (45). Notably, PI3 was found to be suppressed in the acute phase of acute respiratory distress syndrome and to play a critical role in its progression (46). In our study, patients with low PI3 expression in the early stage were at risk of developing IPA. PI3 may be a potential clinical marker for early fungal infection monitoring, with significant implications for improving SFTSV outcomes. In recent years, there has been unparalleled progress in molecular testing platforms at the point of care, especially due to the widespread adoption of these systems during the COVID-19 pandemic (47). Innovations in microfluidics and biosensing technology have greatly enhanced the sensitivity, specificity, and multiplexing capabilities of emerging point-of-care tests, offering hope for future nucleic acid testing platforms (48).

However, there are several limitations to our study. Firstly, it was conducted in a patient population with a high pre-test probability of SFTS, which may limit the generalizability of the findings to broader populations or settings with lower pre-test probabilities. Since the two-transcript signature reflects a general antiviral host response rather than being specific to SFTS, test specificity may be reduced in the presence of other circulating viruses, such as hantavirus. Secondly, the validation cohorts included disproportionately few patients with non-SFTS viral infections, noninfectious conditions, and other types of infections. However, during the study period, other infections such as dengue, malaria, and hantavirus were relatively rare in Shandong Province. Scrub typhus often has a characteristic eschar, making it relatively easier to differentiate from SFTS. These seasonal and geographical limitations may have introduced bias into the study’s findings, limiting the ability to fully assess the differentiation utility of the two-transcript test in more diverse clinical settings. Furthermore, the use of PCR-based methods for diagnosing SFTS may underestimate the actual prevalence of the disease. Finally, due to immune suppression, SFTS patients are highly susceptible to secondary infections. Although chest X-rays, CT scans, sputum microbiology, and GM tests were performed at admission, negative results do not entirely rule out the possibility of SFTS co-infection with IPA.

### Conclusions

In this study, we evaluated the effectiveness of a two-transcript signature in differentiating and identifying severe SFTS cases for during the peak of the SFTS outbreak. Our findings reveal that this signature serves as a valuable biomarker for distinguishing SFTSV infection from bacterial sepsis and aids in risk stratification among SFTS patients. While it does not replace pathogen detection, the two-transcript signature enhances patient triage and effectively identifies high-risk individuals, thereby guiding appropriate treatment decisions and improving prognostic assessments during SFTS epidemic seasons.

## Data Availability

Raw data are available through the corresponding author upon reasonable request.

## SUPPLEMENTAL MATERIAL

Supplemental material jcm.01282-24-s0001.docx Supplemental methods, Fig. S1 to S3, and Tables S1 to S5.

ASM does not own the copyrights to Supplemental Material that may be linked to, or accessed through, an article. The authors have granted ASM a non-exclusive, world-wide license to publish the Supplemental Material files. Please contact the corresponding author directly for reuse.
