# Supplementary material for: Two-transcript signature for differentiation and clinical outcomes in severe fever with thrombocytopenia syndrome (SFTS) patients: a double-blind, multicenter, validation study
Source: J Clin Microbiol. 2024 Dec 17;63(1):e01282-24. doi: 10.1128/jcm.01282-24 (PMC11784442; doi:10.1128/jcm.01282-24)
Supplement: Supplemental material — Supplemental methods, Fig. S1 to S3, and Tables S1 to S5. [file jcm.01282-24-s0001.docx]

**Table of Contents**

**SUPPLEMENTARY METHODS .......................................................................................................2**

**Sample size.............................................................................................................................................2**

**The derivation of the two-transcript regression model......................................................................2**

**RT-PCR assay for the two-transcript signature in the prospective validation cohort......................................................................................................................................................3**

**SUPPLEMENTARY FIGURES ..........................................................................................................5**

**Supplementary Figure 1: Categorization algorithm for the prospective validation cohort...........5**

**Supplementary Figure 2：Performance of the two-transcript signature, C-reactive protein and procalcitonin in this prospective validation cohort.........................................................................6**

**Supplementary Figure 3. The relationship** **between the relative expression of IFI44L, PI3 and viral load.........................................................................................................................................8**

**SUPPLEMENTARY TABLES..............................................................................................................9**

**Supplementary Table 1: Baseline demographics and clinical characteristics in patients with SFTS and patients with bacterial infections....................................................................................9**

**Supplementary Table 2: Measures of diagnostic accuracy of the two-transcript signature for identification of SFTS ...........................................................................................................................10**

**Supplementary Table 3: Baseline demographics and clinical characteristics in fatal SFTS patients and nonfatal SFTS patients................................................................................................11**

**Supplementary Table 4: Baseline demographics and clinical characteristics in SFTS patients with and without invasive pulmonary aspergillosis.......................................................................12**

**Supplementary** **Table 5：Results for the binary logistic regression model.....................................13**

**References..............................................................................................................................................14**

**Sample size**

We anticipate that the area under the receiver operating characteristic curve (AUC) will be at least 0.90, given the AUC of 0.969 achieved by the two-transcript signature in diagnosing bacterial and viral infections in our previous study (1). Assuming a null hypothesis value of 0.7, and referring to the 0.730 AUC of procalcitonin for distinguishing bacterial from viral infections (2), we set a significance level of 1% (α = 0.01) and power of 90% (1-β). We plan to use equal sample sizes for both bacterial and viral infection groups.

Using Medcalc (version 18.2.1) software, we calculated that we need to include 39 patients with bacterial infections and 39 patients with SFTSV infections, totaling 78 subjects. Considering a 10% exclusion rate for technical reasons (such as suboptimal RNA quantity or RT-PCR failure), we require 86 cases. Therefore, we decided to include the entire unselected cohort from a two-year SFTS peak period (May-September).

The bacterial infection likelihood score was calculated according to the relative level of the 2 transcripts in the discovery group (bacterial, n=55; viral, n=69) using a logistic regression algorithm (Figure 3 and Supplementary Table9)

**The derivation of the two-transcript regression model**

In our previous study(1), we used binary logistic regression to combine the expression levels of *IFI44L* and *PI3* into a single score, providing a probabilistic interpretation of bacterial infection by assigning a likelihood score to the patient's diagnosis.

The model development process is as follows: the Ct values of *IFI44L* and *PI3* were used as independent variables, while bacterial infection status (present or absent) served as the dependent variable. The data for model training included 124 cases from the discovery group, consisting of 55 bacterial infection cases and 69 viral infection cases. The model assumptions were verified, with the dependent variable being binary, independent observations, and no collinearity between independent variables. The model achieved an AUC of 0.990 (95% CI 0.978-1.000) for distinguishing bacterial from viral infection. The coefficients and standard errors of the two independent variables in the regression equation are provided in Supplementary Table 5.

**RT-PCR assay for the two-transcript signature in the prospective validation cohort**

TaqMan® Gene Expression assays were purchased from Applied Biosystems targeting *IFI44L* (Hs00915292_m1), *PI3* (Hs00160066_m1), and the internal control *ACTB* (Hs01060665_g1). The experimental workflow consisted of 4 steps: RNA extraction, reverse transcription, gene expression, and data analysis as follows:

**RNA extraction**

Total RNA was extracted using the PAXgene Blood RNA kit according to the manufacturer’s protocol. An ultraviolet spectrophotometer (Nandrop1000, Thermo Fisher) was employed to measure the purity and quantity of the isolated RNA. The extracted RNA was considered suitable if the A260/A280 ratio was >1.6.

**Reverse transcription**

All extracted RNA samples were reverse transcribed into cDNA using a reverse-transcription kit (Catalog#B532435, Sangon Biotech Corp) according to the manufacturer’s instructions. 300 ng RNA was added to a 20-μl mixture containing Random Primer p(dN)6, reaction buffer, RNase Inhibitor, dNTP Mix, M-MuLV RT, and RNase free ddH2O with the following cycling profile: 65°C for 5 min, 2 min in an ice bath, 25°C for 10 min, 50°C for 30 min, and 85°C for 5 min and a hold at 4°C. The obtained cDNA was stored at -80°C for further detection.

**Real-time PCR**

Reactions were performed in a 20-ul reaction volume in TaqMan Fast qPCR Master Mix (Catalog# B639276, Sangon Biotech Corp) using Hongshi SLAN96P platform. Single-gene single-channel dual-repetitive amplification was performed using a fluorescent quantitation PCR instrument. The PCR cycle was 50°C for 2 min, 95°C for 10 min and 36 cycles of 95°C for 15 s and 60°C for 1 min.

**Data analysis**

All samples were assayed by duplicate, and the average was used. The relative expression levels of IFI44L and PI3 was evaluated using the delta Ct method (3) , ACTB as the internal control : delta Ct = (Ct gene of interest- Ct internal control). The Ct is defined as the PCR cycle at which the fluorescent signal of the reporter dye crosses the threshold. C_t_ gene of interest, C_t_ internal control used here were the mean of these PCR replicates. The mean Ct value of IFI44L and PI3 were directly brought into the 2-transcripts logistic regression model and calculated the bacterial infection likelihood score. The coefficients and standard errors of the two independent variables (IFI44L and PI3) in the regression equation see Supplementary Table 5. The cutoff of the bacterial infection likelihood score was 0.547598; the larger the value is, the more likely the case is bacterial infection; below 0.547598 were classified as viral, the smaller the value, the higher the likelihood of a viral infection. All modeling analyses were carried out using SPSS 24.0 (IBM Corp., Somers, NY, USA) statistical software.

**SUPPLEMENTARY FIGURES**

*Coagulase-negative Staphylococcus bacteraemia is considered as contamination and not as infection; ^#^Diagnoses of infectious mononucleosis and epidemic haemorrhagic fever were based on the specific IgM of the corresponding virus.

**Supplementary Figure 1. Categorization algorithm for the prospective validation cohort.**

Algorithm used to categorize cases for the prospective validation cohort into bacterial, SFTS, Non- SFTS viral and unknown (lacking diagnostic certainty) groups. Noninfectious cases and other infections were categorized separately.


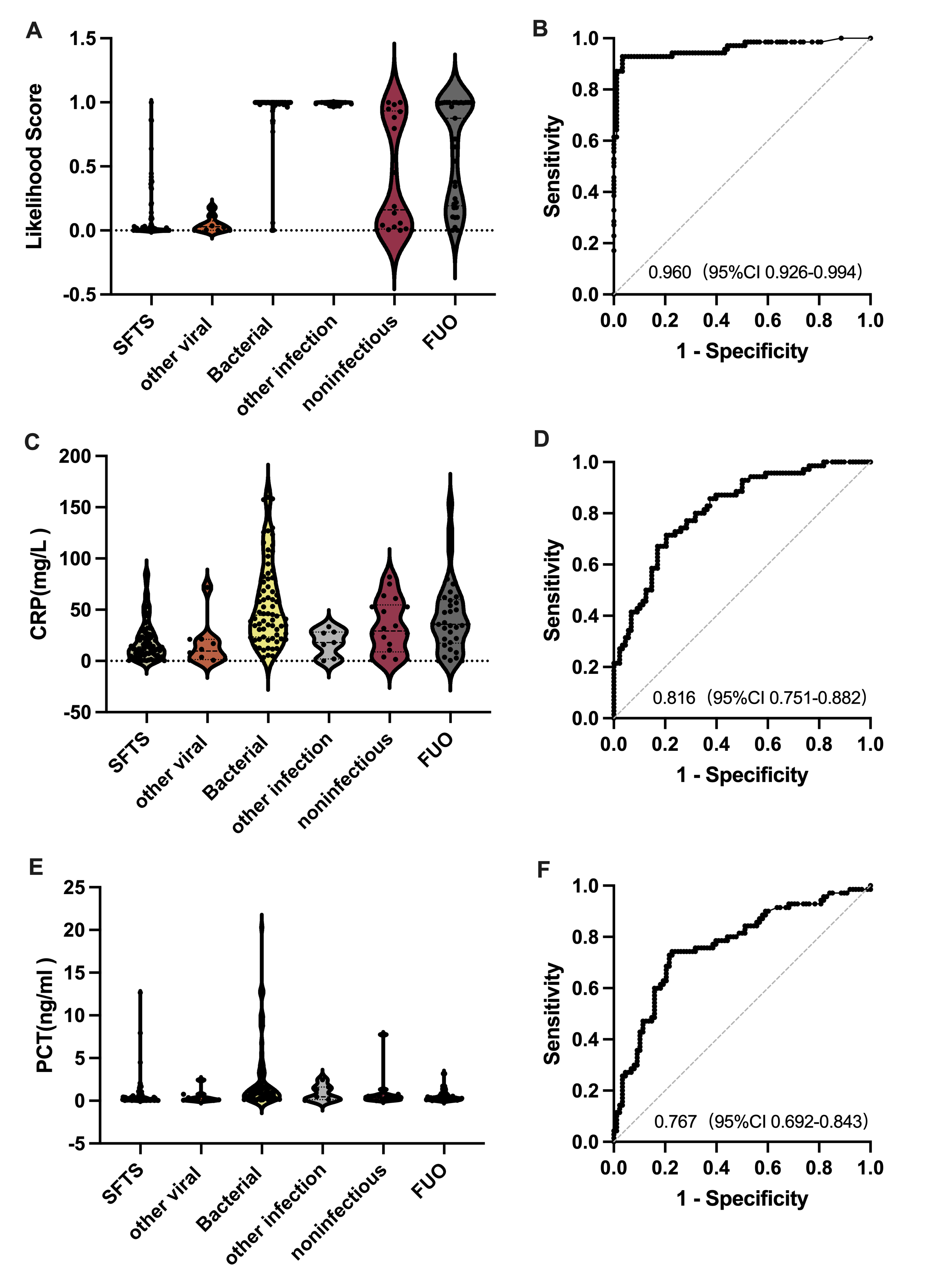


**Supplementary Figure 2. Performance of the two-transcript signature, C-reactive protein and procalcitonin in this prospective validation cohort.**

Boxplots displaying the two-transcript signature (A), CRP (C), and PCT (E) in the prospective validation cohort, comparing different categories: bacterial, SFTS, non-SFTS viral, other infections, noninfectious disease and fever of unknown origin (lacking diagnostic certainty). ROC curves of the two-transcript signature (B), CRP (D), and PCT (F) differentiate between bacterial and viral infections (combining the SFTS group with other viral infections). Boxplots show the means and IQRs. AUC, area under the curve; CRP, C-reactive protein; PCT, procalcitonin; ROC, receiver operating characteristic; SFTS, severe fever with thrombocytopenia syndrome; FUO, fever of unknown origin; ****indicates a p value <0.0001.


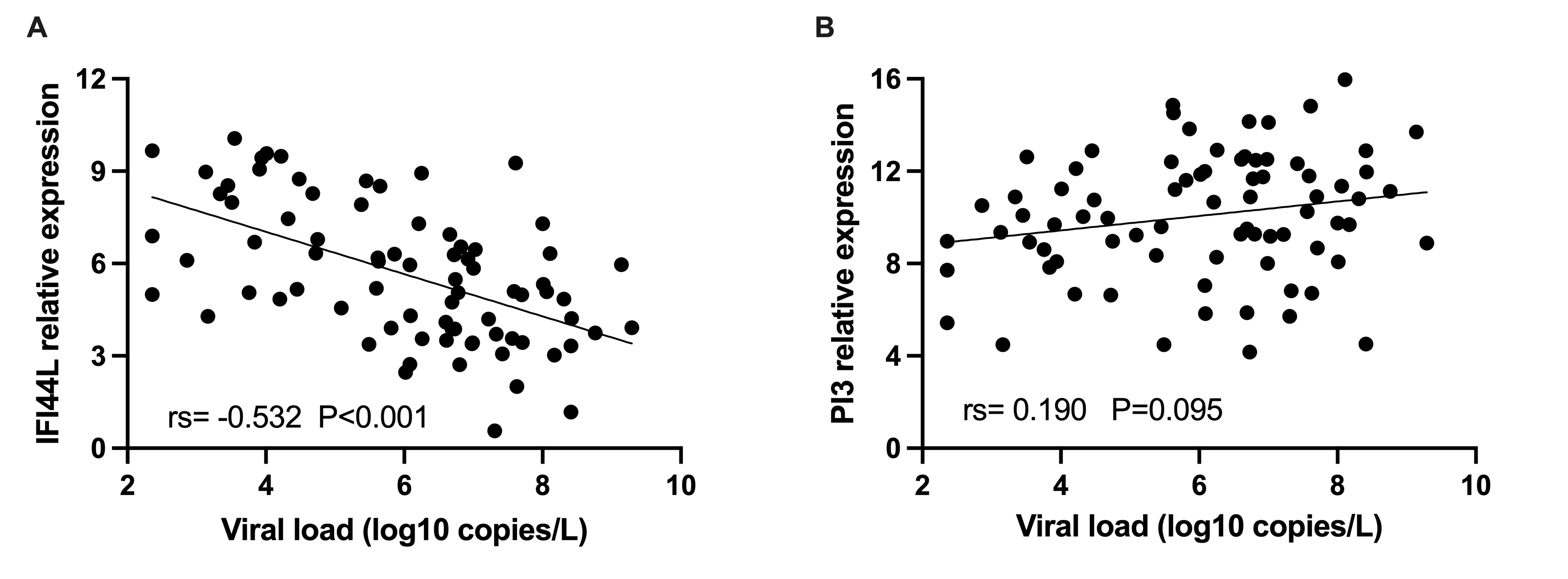


**Supplementary Figure 3. The relationship** **between the relative expression of IFI44L, PI3 and viral load**

The relative expression of IFI44L and PI3 were displayed using delta Ct values, while SFTSV RNA concentrations (copies/mL) were log-transformed. A scatter plot illustrates this relationship, with each point representing an observation. In plot (A), the horizontal axis represents the relative expression of IFI44L, while the vertical axis shows the SFTSV viral load, highlighting a positive correlation between IFI44L expression and viral load. In plot (B), the horizontal axis represents the relative expression of PI3, while the vertical axis shows the SFTSV viral load, highlighting a negative correlation between PI3 expression and viral load.

**SUPPLEMENTARY TABLES**

**Supplementary Table 1: Baseline demographics and clinical characteristics in patients with SFTS and patients with bacterial infections**

|  | **Reference Range** | **Total**  **(n=148)** | **SFTS**  **(n=78)** | **Bacterial**  **(n=70)** | **P value** |
| --- | --- | --- | --- | --- | --- |
| **Patient characteristics** |  |  |  |  |  |
| Age, years |  | 66 (59-73) | 66 (60-73) | 66 (57-72) | 0.568 |
| Sex |  |  |  |  | 0.149 |
| Male |  | 86 (58%) | 41 (53%) | 45 (64%) |  |
| Female |  | 62 (42%) | 37 (47%) | 25 (36%) |  |
| Duration of symptoms, days |  | 8 (6-10) | 7 (6-9) | 8 (6-10) | 0.255 |
| **Clinical metrics** |  |  |  |  |  |
| WBC (x10^9^ /L) | 3.5-9.5 | 3.48 (2.34-4.94) | 3.03 (1.86-4.97) | 3.99 (2.86-4.96) | 0.022 |
| NEU (x10^9^ /L) | 1.8-6.3 | 2.46 (1.43-3.81) | 1.75 (0.99-2.85) | 3.04 (2.24-4.12) | <0.001 |
| LYM (x10^9^ /L) | 1.1-3.2 | 0.57 (0.32-1.00） | 0.71 (0.43 -1.19) | 0.41 (0.24-0.74) | <0.001 |
| Hemoglobin (g/l) | 130-175 | 130 (108-146) | 139 (128-154) | 117 (102-132) | <0.001 |
| Platelet (x109 /L) | 125-350 | 60 (40-79) | 60 (41-81) | 59 (39-79) | 0.840 |
| CRP (mg/L) | 0-10 | 25.19（10.67-53.22） | 13.59 (5.59-27.63) | 44.63 (23.35-80.30) | <0.001 |
| PCT (ng/ml) | < 0.1 | 0.382（0.130-1.314） | 0.198（0.089-0.423） | 0.946 (0.320-2.823) | <0.001 |
| Ferritin (ng/ml) | 13-400 | 2008 (843-4857) | 3940 (1752-8439) | 1044 (675-2121) | <0.001 |
| ALT(U/L) | 9-50 | 65 (30-145) | 75 (44-143) | 46 (24-159) | 0.053 |
| AST(U/L) | 15-40 | 112 (40-240) | 139 (83-398) | 52 (24-141) | <0.001 |
| Albumin (g/L) | 40-55 | 32 (29-34) | 33 (30-35) | 30 (28-33) | 0.029 |
| LDH (mmol/L) | 120-230 | 599 (358-979) | 736 (511-1181) | 486 (268-737) | <0.001 |
| Cr (μmol/L) | 62-115 | 65 (51-93) | 65 (51-87) | 65 (52-112) | 0.661 |
| BUN (mmol/L) | 2.3-7.8 | 6.1 (4.3-9.9) | 5.5 (4.3-8.3) | 7.3 (4.3-14.6) | 0.025 |
| PT (second) | 11-14.5 | 12.9 (11.9-14.3) | 12.5 (11.3-13.3) | 13.8 (12.5-15.5) | <0.001 |
| APTT (second) | 28.0-45.0 | 39.1 (32.7-47.2) | 41.3 (33.0-50.5) | 37.3 (32.6-43.4) | 0.048 |
| Fibrinogen(g/l) | 2.0-4.0 | 3.35 (2.47-4.88) | 2.74 (2.33-3.61) | 4.14 (3.09-5.45) | <0.001 |
| D-dimer (μg/ml) | < 0.5 | 2.41 (1.19-4.72) | 1.96 (1.06-4.29) | 2.60 (1.29-6.11) | 0.133 |

All data are given as n (%) and median (inter-quartile range). Continuous variables were compared using the Mann–Whitney U test. Categorical variables were compared using the Fisher’s exact test or chi square test, where appropriate.

Abbreviations: SFTS, severe fever with thrombocytopenia syndrome; WBC, white blood count; NEU, neutrophils; LYM, lymphocytes; CRP, C-reactive protein; PCT, procalcitonin; AST, aspartate aminotransferase; ALT, alanine aminotransferase; LDH, lactic dehydrogenase; BUN, blood urea nitrogen; Cr, creatinine; PT, prothrombin time; PT, prothrombin time; APTT: activated partial thromboplastin time

**Supplementary Table 2: Measures of diagnostic accuracy of the 2-transcripts signature for identification of SFTS**

|  | **2-transcripts** | **CRP** | **PCT** |
| --- | --- | --- | --- |
| AUC (95%CI) | 0.961 (0.916-0.986) | 0.810 (0.738-0.870) | 0.764 (0.687-0.830) |
| Sensitivity (95%CI) | 0.929 (0.843- 0.969) | 0.714 (0.600−0.807) | 0.714 (0.560−0.807) |
| Specificity (95%CI) | 0.949 (0.875-0.980) | 0.769 (0.664−0.849) | 0.782 (0.678−0.859) |
| PPV (95%CI) | 0.942 (0.860-0.977) | 0.735 (0.620-0.826) | 0.746 (0.631-0.835) |
| NPV (95%CI) | 0.937 (0.860-0.973) | 0.750 (0.645-0.832) | 0.753 (0.649-0.834) |
| LR+ (95%CI) | 18.110 (11.070-29.630) | 3.095 (2.733-3.506) | 3.277 (2.875-3.736) |
| LR- (95% CI) | 0.075 (0.051-0.112) | 0.371 (0.334-0.414) | 0.365 (0.328-0.407) |
| Accuracy (95% CI) | 0.939 (0.889-0.968) | 0.743 (0.667-0.807) | 0.750 (0.675-0.813) |

A likelihood Score cutoff of 0.547598, a PCT cutoff of 0.5 ng/ml and a CRP cutoff of 30 mg/l were used to calculate accuracy, sensitivity, specificity, positive predictive value, negative predictive value, positive likelihood ratio, and negative likelihood ratio. Data are point estimates (95% CIs).

Abbreviations: SFTS, severe fever with thrombocytopenia syndrome; CRP, C-reactive protein; PCT, procalcitonin; AUC, area under the receiver operating characteristic curve; CI, Confidence interval, PPV, positive predictive value; NPV, negative predictive value; LR+, positive likelihood ratio; LR-, negative likelihood ratio.

**Supplementary Table 3: Baseline demographics and clinical characteristics in fatal SFTS patients and nonfatal SFTS patients**

|  | **Reference Range** | **Total**  **(n=78)** | **Nonfatal**  **(n=60)** | **Fatal**  **(n=18)** | **P value** |
| --- | --- | --- | --- | --- | --- |
| **Patient characteristics** |  |  |  |  |  |
| Age, years |  | 66 (60-73) | 66 (59-72) | 69 (62-75) | 0.156 |
| Sex |  |  |  |  | 0.804 |
| Male |  | 41 (53%) | 32 (53%) | 9 (50.0%) |  |
| Female |  | 37 (47%) | 28 (47%) | 9 (50.0%) |  |
| Duration of symptoms, days |  | 7 (6-9) | 7 (5-10) | 8 (7-9) | 0.319 |
| **Clinical metrics** |  |  |  |  |  |
| WBC (x10^9^ /L) | 3.5-9.5 | 3.03 (1.86-4.97) | 2.92 (1.80-4.63) | 3.86 (2.49-7.51) | 0.115 |
| NEU (x10^9^ /L) | 1.8-6.3 | 1.75 (0.99-2.85) | 1.65 (0.86-2.59) | 2.43 (1.63-5.53) | 0.025 |
| LYM (x10^9^ /L) | 1.1-3.2 | 0.71 (0.43-1.19) | 0.67 (0.42-1.23) | 0.77 (0.48-1.10) | 0.565 |
| Hemoglobin (g/l) | 130-175 | 139 (128-154) | 138 (126-154) | 147 (132-154) | 0.444 |
| Platelet (x10^9^ /L) | 125-350 | 60 (41-81) | 62 (40-82) | 50 (40-68) | 0.206 |
| CRP (mg/L) | 0-10 | 13.59 (5.59-27.63) | 10.73 (4.26-21.33) | 41.29 (17.40-56.66) | <0.001 |
| PCT (ng/ml) | < 0.1 | 0.198 (0.089-0.423) | 0.132（0.080-0.318） | 1.012 (0.233-1.722) | <0.001 |
| Ferritin (ng/ml) | 13-400 | 3940 (1752-8439) | 2933 (1340-4922) | 8467 (5463-11538) | <0.001 |
| ALT(U/L) | 9-50 | 75 (44-143) | 67 (35-142) | 94 (66-160) | 0.070 |
| AST(U/L) | 15-40 | 139 (83-398) | 122 (79-293) | 360 (150-634) | 0.001 |
| Albumin (g/L) | 40-55 | 33 (30-35) | 33 (30-36) | 31 (27-33) | 0.029 |
| LDH (mmol/L) | 120-230 | 736 (511-1181) | 615 (469-1023) | 1094 (903-1646) | 0.001 |
| Cr (μmol/L) | 62-115 | 65 (51-87) | 63 (49-77) | 93 (59-136) | 0.001 |
| BUN (mmol/L) | 2.3-7.8 | 5.5 (4.3-8.3) | 5.1 (4.1-6.6) | 9.9 (5.7-15.1) | <0.001 |
| PT (second) | 11-14.5 | 12.5 (11.3-13.3) | 12.5 (11.1-13.1) | 13.6 (11.6-14.5) | 0.031 |
| APTT (second) | 28.0-45.0 | 41.3 (33.0-50.5) | 39.0 (31.1-47.5) | 49.4 (40.8-60.9) | 0.004 |
| Fibrinogen(g/l) | 2.0-4.0 | 2.74 (2.33-3.61) | 3.01 (2.43-3.58) | 2.44 (1.79-3.74) | 0.063 |
| D-dimer (μg/ml) | < 0.5 | 1.96 (1.06-4.29) | 1.87 (0.93-3.94) | 2.75 (1.56-8.06) | 0.023 |
| Viral loads(log_10_copies/ml) |  | 6.26 (4.47-7.35) | 5.84 (4.06-6.90) | 7.65 (6.22-8.41) | <0.001 |

Abbreviations: SFTS, severe fever with thrombocytopenia syndrome; WBC, white blood count; NEU, neutrophils; LYM, lymphocytes; CRP, C-reactive protein; PCT, procalcitonin; AST, aspartate aminotransferase; ALT, alanine aminotransferase; LDH, lactic dehydrogenase; BUN, blood urea nitrogen; Cr, creatinine; PT, prothrombin time; PT, prothrombin time; APTT: activated partial thromboplastin time

**Supplementary Table 4: Baseline demographics and clinical characteristics in SFTS patients with and without invasive pulmonary aspergillosis**

|  | **Reference Range** | **Total**  **(n=78)** | **with IPA**  **(n=39)** | **without IPA**  **(n=39)** | **P value** |
| --- | --- | --- | --- | --- | --- |
| **Patient characteristics** |  |  |  |  |  |
| Age, years |  | 66 (60-73) | 67 (62-74) | 66 (59-72) | 0.545 |
| Sex |  |  |  |  | 0.496 |
| Male |  | 41 (53%) | 22 (56%) | 19 (49%) |  |
| Female |  | 37 (47%) | 17 (44%) | 20 (51%) |  |
| Duration of symptoms, days |  | 7 (6-9) | 8 (7-9) | 7 (5-10) | 0.293 |
| **Clinical metrics** |  |  |  |  |  |
| WBC (x10^9^ /L) | 3.5-9.5 | 3.03 (1.86-4.97) | 3.78 (1.80-8.48) | 2.72 (1.87-3.64) | 0.048 |
| NEU (x10^9^ /L) | 1.8-6.3 | 1.75 (0.99-2.85) | 2.29 (1.12-6.44) | 1.65 (0.84-2.10) | 0.015 |
| LYM (x10^9^ /L) | 1.1-3.2 | 0.71 (0.43-1.19) | 0.71 (0.48-1.24) | 0.65 (0.42-1.12) | 0.433 |
| Hemoglobin (g/l) | 130-175 | 139 (128-154) | 139 (125-154) | 139 (129-154) | 0.719 |
| Platelet (x10^9^ /L) | 125-350 | 60 (41-81) | 43 (31-70) | 67 (58-87) | <0.001 |
| CRP (mg/L) | 0-10 | 13.59 (5.59-27.63) | 22.98 (13.51-38.39) | 7.29 (1.25-14.17) | <0.001 |
| PCT (ng/ml) | < 0.1 | 0.198（0.089-0.423） | 0.330 (0.151-1.092) | 0.120 (0.059-0.264) | <0.001 |
| Ferritin (ng/ml) | 13-400 | 3940 (1752-8439) | 5874 (3169-10979) | 2587 (1145-4563) | <0.001 |
| ALT(U/L) | 9-50 | 75 (44-143) | 96 (59-154) | 57 (31-115) | 0.004 |
| AST(U/L) | 15-40 | 139 (83-398) | 265 (128-480) | 108 (63-189) | <0.001 |
| Albumin (g/L) | 40-55 | 33 (30-35) | 31(27-34) | 34 (31-36) | 0.002 |
| LDH (mmol/L) | 120-230 | 736 (511-1181) | 1098 (693-1609) | 564 (460-752) | <0.001 |
| Cr (μmol/L) | 62-115 | 65 (51-87) | 73 (55-97) | 62 (49-77) | 0.028 |
| BUN (mmol/L) | 2.3-7.8 | 5.5 (4.3-8.3) | 6.8 (4.9-9.9) | 4.7 (3.5-6.1) | <0.001 |
| PT (second) | 11-14.5 | 12.5 (11.3-13.3) | 12.6 (10.8-13.7) | 12.4 (11.5-13.1) | 0.603 |
| APTT (second) | 28.0-45.0 | 41.3 (33.0-50.5) | 46.1 (39.0-55.5） | 35.0 (30.9-47.2) | 0.007 |
| Fibrinogen(g/l) | 2.0-4.0 | 2.74 (2.33-3.61) | 3.07 (2.28-3.90） | 2.60 (2.35-3.26) | 0.360 |
| D-dimer (μg/ml) | < 0.5 | 1.96 (1.06-4.29) | 1.87 (1.10-4.73) | 2.30 (1.06-4.02) | 0.667 |
| Viral loads(log_10_copies/ml) |  | 6.26 (4.47-7.35) | 7.03 (6.02-8.00) | 5.62 (3.84-6.72) | <0.001 |

Abbreviations: SFTS, severe fever with thrombocytopenia syndrome; IPA, invasive pulmonary aspergillosis, WBC, white blood count; NEU, neutrophils; LYM, lymphocytes; CRP, C-reactive protein; PCT, procalcitonin; AST, aspartate aminotransferase; ALT, alanine aminotransferase; LDH, lactic dehydrogenase; BUN, blood urea nitrogen; Cr, creatinine; PT, prothrombin time; PT, prothrombin time; APTT: activated partial thromboplastin time

**Supplementary** **Table 5： Results for the binary logistic regression model.**

| **Variable** | **Coefficient** | **S.E** |
| --- | --- | --- |
| *IFI44L* | -0.831 | 0.326 |
| *PI3* | 1.208 | 0.527 |
| Constant | -3.867 | 4.579 |

Abbreviations: S.E, Standard Error

**References**

1. Xu N, Hao F, Dong X, Yao Y, Guan Y, Yang L, Chen F, Zheng F, Li Q, Liu W, Zhao C, Li W, Palavecino E, Wang W, Wang G. 2021. A two-transcript biomarker of host classifier genes for discrimination of bacterial from viral infection in acute febrile illness: a multicentre discovery and validation study. Lancet Digit Health 3:e507-e516.

2. Kamat IS, Ramachandran V, Eswaran H, Guffey D, Musher DM. 2020. Procalcitonin to Distinguish Viral From Bacterial Pneumonia: A Systematic Review and Meta-analysis. Clin Infect Dis 70:538-542.

3. Schmittgen TD, Livak KJ. 2008. Analyzing real-time PCR data by the comparative C(T) method. Nat Protoc 3:1101-8.
